# Residues Analysis and Dissipation Dynamics of Broflanilide in Rice and Its Related Environmental Samples

**DOI:** 10.1155/2020/8845387

**Published:** 2020-12-15

**Authors:** Guai Xie, Wenwen Zhou, Mingxia Jin, Ailin Yu, Lei Rao, Horan Jia, Juan Luo, Yichang He, Baotong Li

**Affiliations:** ^1^College of Agriculture, Jiangxi Agricultural University, 1225 Zhimin Road, Economic and Technological Development Area, Nanchang, China; ^2^Jiangxi Academy of Forestry, 1629 Fenglin Road, Economic and Technological Development Area, Nanchang, China; ^3^Jiangxi GuYao Agricultural Science and Technology Co. Ltd, 1388 Jingkai Road, Economic and Technological Development Area, Nanchang, China; ^4^School of Food, Jiangxi Agricultural University, 1225 Zhimin Road, Economic and Technological Development Area, Nanchang, China

## Abstract

Herein, we present a method for the quantitative analysis of broflanilide residues in water, soil, and rice samples from a paddy field in Jiangxi Province, China. The quick, easy, cheap, effective, rugged, and safe (QuEChERS) method was optimized for the extraction and purification of broflanilide residues. Residual broflanilide concentrations in different matrices were then determined by high-performance liquid chromatography (HPLC). The calibration curve of broflanilide showed good linearity in all matrices for concentrations between 0.005 and 1 mg·L^−1^, with a correlation coefficient greater than 0.99. The matrix effect varied from −69% to −54%, indicating matrix suppression. The average recoveries ranged between 85.82% and 97.46%, with relative standard deviations of 3.29%–8.15%. The limits of detection ranged from 0.16 to 1.67 *μ*g·kg^−1^, and the limits of quantification were in the range of 0.54 to 5.48 *μ*g·kg^−1^. Dissipation dynamic tests indicated broflanilide half-lives of 0.46–2.46, 2.09–5.34, and 1.31–3.32 days in soil, water, and rice straw, respectively. Broflanilide was dissipated more rapidly in water than in soil and rice straw. More than 90% of broflanilide residues dissipated within 14 days. The final residues of broflanilide in rice were all below LOQ at harvest.

## 1. Introduction

Rice (*Oryza sativa* L.) is one of the world's most important grain crops. Since the 1990s, global rice production has grown at an annual rate of 0.4%, and the average yield has increased annually by ∼2% [1, 2]. In China, rice accounts for a large proportion of food production and therefore plays a critical role in the nation's economic development [3]. In recent decades, pests have posed an increasing challenge to rice production mainly due to the indiscriminate use of pesticides, changes in cropping systems, and rising global temperatures [4]. Frequent outbreaks of pests can reduce yields and even cause rice crops to fail, which thus pose a major threat to food security and safety [5]. Minimizing the spread of pests and improving the yield and quality of rice grains has thus become an urgent task [6]. In this context, copious quantities of insecticides are widely used for the prevention and control of pests in paddy fields, and the resulting residues of pesticides in media such as soil, water, and rice pose a threat to human health [7]. Sensitive and accurate detection methods for residual levels and dissipation of pesticides are therefore needed, which could facilitate pollution control and ensure food and environmental safety [8].

**Figure 1:**
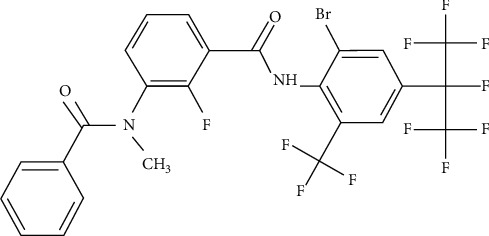
Chemical structural formula of broflanilide.

Broflanilide, [3-benzamido-N-(4-(perfluoropropan-2-yl)phenyl)benzamide] (Figure 1), is a new type of diamide insecticide jointly developed by Mitsui Chemicals Agro, Inc. (Tokyo, Japan) and BASF Corp. (Ludwigshafen, Germany). It exhibits high larvicidal activity against caterpillars, beetles, termites, and other common pests on leafy vegetables, cereals, and perennial crops [9]. Evidence suggests that broflanilide is metabolized to desmethyl-broflanilide, which acts as a noncompetitive resistant-to-dieldrin *γ*-aminobutyric acid receptor antagonist. The binding site of desmethyl-broflanilide differs from that of conventional noncompetitive antagonists such as fipronil [10]. Hence, broflanilide may have a prominent position in the future and could be extensively used for controlling pests with resistance to noncompetitive antagonists [11]. Prior studies have explored the physical and chemical properties of broflanilide. To the best of our knowledge, there is just one study from a Chinese agricultural university which investigated the determination of broflanilide in five typical Chinese soils by high-performance liquid chromatography (HPLC) [12], but this study has not researched the broflanilide residues in paddy water, rice straw, rice husks, and rice grains. Furthermore, the dissipation dynamics of broflanilide residues in related environmental samples has not been ever studied yet. To control the possible effects of broflanilide on human health and food safety, it is critical to establish an effective analytical method for the detection of broflanilide residues in rice and related environmental matrices.

In recent years, high-performance liquid chromatography (HPLC) combined with the quick, easy, cheap, effective, rugged, and safe (QuEChERS) method has been extensively applied for the detection of pesticide residues in food and environmental samples [13]. HPLC has proved to be a powerful technique with high sensitivity, selectivity, and precision [14]. Particularly, it is superior to gas chromatography in the separation and detection of target compounds with high boiling point and low volatility [15]. When preparing samples for insecticide residue analysis, conventional methods such as liquid-liquid and liquid-solid extraction require a substantial amount of organic solvent and involve complicated operation procedures; compared to other methods, such as SPME and MSPD, the QuEChERS method is suitable for many pesticides and has higher recoveries. Furthermore, the QuEChERS method is more economical in both time and labor and more environmentally friendly because it consumes less solvent [16, 17]. However, HPLC combined with the QuEChERS method has not yet been reported for the determination of broflanilide residues in rice and related environmental samples.

The present study proposes a simple and reliable method to determine broflanilide residues in rice and related environmental samples collected from a paddy field, and the dissipation dynamics of broflanilide in rice straw, paddy water, and paddy soil were investigated as well to study the ultimate residue under Good Agricultural Practices use. Up to now, no residue limit has been established on broflanilide in rice in China. The residue data obtained from this study would be useful in establishing maximum residue limit (MRL) and dietary intake assessment. These results could guide the development of analytical methods to determine diamide insecticide residues in rice and related environments.

## 2. Materials and Methods

### 2.1. Reagents and Materials

Broflanilide standard (≥98.6%) and concentrate suspension (5%) were purchased from Mitsui Chemicals Agro Inc. (Tokyo, Japan). Chromatographically pure formic acid (≥88%) was purchased from Komio Chemical Reagent Co., Ltd. (Tianjin, China). Chromatographic grade methanol, acetonitrile, sodium chloride (NaCl), and anhydrous magnesium sulfate (MgSO_4_) were bought from Xilong Scientific Co., Ltd. (Shantou, China). Graphitized carbon black (GCB) (60 *μ*m) was provided by XFNANO Materials Tech Co., Ltd. (Nanjing, China). *N*-propylethylenediamine (PSA) (40 *μ*m) and octadecylsilane (C18) (40 *μ*m) were provided by Bonna-Agela Technologies (Tianjin, China). Ultrapure water was obtained from a Milli-Q water purification system (Millipore Corp., Billerica, USA).

### 2.2. Preparation of Standard Solutions

A 101.4 mg sample of the broflanilide standard (accurate to 0.0002 g) was weighed into a 100 mL volumetric flask using a Mettler Toledo AL204 analytical balance (±0.0001 g; Shen Borui Instrument Co., Ltd, Shenzhen, China). The standard sample was dissolved with 85 mL of acetonitrile under ultrasonication for 20 min using a SB-5200DT ultrasonic cleaner (Xinzhi Biotechnology Co., Ltd., Ningbo, China). The solution was allowed to sit at room temperature for 40 min. Then the stock solution containing 1 mg·mL^−1^ broflanilide was shaken and kept at 3°C until used. Working solutions were freshly prepared by diluting the stock solution with acetonitrile to give gradient concentrations of broflanilide (0.05, 0.5, 1, 2, 5, 10, 20, and 50 mg·L^−1^). To prepare matrix standard solutions, blank matrix solutions obtained after sample pretreatment were weighed and mixed with the working solutions.

### 2.3. Field Experiment Design

The field experiments were conducted in two consecutive years in July (2018 and 2019) at Zengjia Village, Yichun city (Jiangxi, China). The dissipation experiments and final residue experiments in supervised field trials were designed according to the pesticide label (provisionally registered by the Chinese government) and the “Guideline for Pesticide Residue Trials” issued by the Institute of the Control of Agrochemicals, Ministry of Agriculture, People's Republic of China (NY/T 788-2018) [18]. The area of the experiment plot was 30 m^2^ (5 × 6 m) and each treatment had three replicated plots. Blank plots were set as a contrast. Every plot was separated by an irrigation channel. In the control treatment, no pesticide was used throughout the entire period of rice growth [19].

To investigate dissipation dynamics of broflanilide in rice straw, paddy water, and soil, broflanilide 5% SC were sprayed with a T-HS16D knapsack electric sprayer at the rice tillering stage at an active constituent dose of 45 g a.i·ha^−1^ (1.5-fold higher than the recommended high dosage) [20]. Dissipation samples (rice straw, paddy water, and paddy soil) were collected randomly from each plot at different time intervals, i.e., 2 h and 1, 3, 5, 7, 14, 21, and 45 days after spraying [18]. The level of broflanilide detected at 2 h was considered as the initial residual level.

For the terminal residue field experiment, broflanilide was applied at two dosage levels, 30 g a.i·ha^−1^ (low concentration, recommended dosage) and 45 g a.i·ha^−1^ (high concentration, 1.5-fold higher than the recommended dosage) with one-time spray, respectively. Each treatment had three replicated plots. Final residues samples were collected randomly from each plot at harvest time [21].

### 2.4. Sample Collection and Pretreatment

Blank samples were taken from an experimental plot (10 m × 6 m) of a paddy field without application of broflanilide in Zengjia Village, Yichun City (Jiangxi, China). The sampling was conducted at the harvest stage of rice. Briefly, five 1 L samples of paddy water were taken at random using a beaker, then mixed and filtered. Similarly, paddy soil (1 kg) was collected from a depth of 0–20 cm in the plot at five random points and thoroughly mixed. After air-drying, the soil sample was passed through a 20-mesh sieve, and 500 g of subsamples were obtained by the quadruple method for extraction [22]. Five 1 kg samples of rice straw were also collected at random, cut into 0.5 cm lengths, then mixed and crushed using an FW80 high-speed universal crusher (Yongguangming Medical Instrument Factory, Beijing, China). During the mature stage, five 1 kg samples of rice panicle were collected at random and shelled; the rice husks and grains were then crushed separately. All rice samples were quartered to 500 g subsamples. The samples were stored at −18°C or lower until used.

Extraction of broflanilide residues from spiked samples was performed using acetonitrile. For paddy water, a 5 mL sample was transferred into a 50 mL centrifuge tube followed by the addition of 10 mL of acetonitrile with 0.1% formic acid. Vigorous vortexing was performed for 5 min using a HY-2 vortex mixer (Leici Instrument Manufacturing Co., Ltd., Shanghai, China). Then 2 g of both MgSO_4_ and NaCl were added to each tube, followed by vigorous vortexing for 2 min. The tube was centrifuged at 11,000 rpm for 5 min using a T15A63 high-speed large-capacity refrigerated centrifuge (Hitachi, Tokyo, Japan). Paddy soil and rice samples (5 g) were extracted following a similar procedure, except that each sample received an appropriate volume of ultrapure water before the addition of acetonitrile with 0.1% formic acid. Specifically, 5 mL of ultrapure water was added to the paddy soil sample, 7 mL was added to the rice grain sample, and 10 mL was added to rice husks and straw samples. For all extractions, 1.5 mL of the supernatant was transferred into a 2.5 mL centrifuge tube containing 150 mg MgSO_4_ and specific sorbents: 50 mg PSA for paddy water, 50 mg C18 for paddy soil and rice grains, and 10 mg GCB + 50 mg C18 for rice straw and husks. The tube was vortexed for 1 min and centrifuged at 5000 rpm for 5 min. The supernatant was filtered through a 0.22 *μ*m organic membrane filter (Keyilong Experimental Equipment Co., Ltd., Tianjin, China), and the filtrate was collected for HPLC analysis.

### 2.5. Chromatographic Instrumentation and Optimal Conditions

HPLC was performed using an Agilent 1260 high-performance liquid chromatograph (Agilent Technologies, Santa Clara, USA) equipped with an autosampler and an ultraviolet (UV) detector. The chromatographic separation was performed on an Agilent Zorbax Eclipse XDB-C18 column (4.6 mm × 150 mm × 5 *μ*m). The mobile phase consisted of 0.1% (v/v) formic acid in water and chromatographic grade acetonitrile (25 : 75, v/v). Other chromatographic conditions were as follows: detection wavelength, 254 nm; column temperature, 40°C; flow rate, 0.5 mL·min^−1^; injection volume, 10 *μ*L.

### 2.6. Method Validation and Data Analysis

Validation of the proposed QuEChERS-HPLC method was performed according to the guidelines of the Collaborative International Pesticides Analytic Council. Specifically, the specificity, linearity, limit of detection (LOD), limit of quantification (LOQ), matrix effect, accuracy, and precision of the method were assessed for different matrices [23, 24].

To evaluate its specificity, the method was applied to blank samples: an absence of interference would indicate that the method is specific for broflanilide. The linearity of the method was determined based on analytical curves established at six different broflanilide concentration levels (0.005, 0.01, 0.05, 0.1, 0.5, and 1.0 mg·L^−1^). The parameters of linear regression equations (correlation coefficient, slope, and intercept) were calculated by least-squares regression. The correlation coefficients and goodness-of-fit were obtained, and values should be ≥ 0.99 and ≤ 20%, respectively [25]. The LOD and LOQ values for spiked samples were calculated based on signal-to-noise ratios of 3 : 1 and 10 : 1, respectively [26].

The matrix effect was determined using acetonitrile as a blank control. For each matrix, a standard solution of broflanilide was prepared according to the concentration gradient, and a standard curve was then constructed. The matrix effect constant was calculated using the following equation [27]:

(1) $$\begin{array}{c} \text{ME}=\left(\frac{S_{m}}{S_{s}}-1\right)\times 100\%, \end{array}$$

where ME is the matrix effect constant; *S*_*m*_ is the slope of the calibration curve in the matrix; *S*_*s*_ is the slope of the calibration curve in the solvent. A ME > 10% indicates matrix enhancement, whereas ME < −10% indicates matrix suppression; values between −10% and 10% mean the matrix effect can be ignored.

The accuracy and precision of the proposed method were tested via recovery experiments. Replicated soil, water, and rice samples were spiked with broflanilide at 0.01, 0.1, and 0.5 mg·kg^−1^ (*n* = 5 for each concentration level) on three different days. To evaluate the accuracy, the recovery (%) of broflanilide in spiked samples was calculated using the calibration curves; values of 70%–110% were acceptable [28]. The relative standard deviation (RSD) of broflanilide recovery was used to evaluate the precision, with values below 20% indicating high precision [29].

To validate the applicability of the proposed method, the method was also applied to samples collected from paddy fields with broflanilide application (30 g a.i·ha^−1^). A total of 100 samples (20 for each matrix) were obtained from different experimental plots of Jiangxi Agricultural University (Nanchang, Jiangxi, China). All samples were pretreated by the optimized QuEChERS procedure and analyzed by HPLC using the optimal conditions.

The dissipation process follows the first-order kinetic reaction. The dissipation rate of broflanilide was expressed in terms of half-life (*T*_1/2_) values, representing the time for the broflanilide level to decrease to half its original value through chemical or biological processes [30]. The *T*_1/2_ values, along with the corresponding dissipation profiles, were obtained by fitting a first-order kinetic equation, which has been extensively used to describe the dissipation processes of various chemicals [31, 32]. The dissipation kinetics of broflanilide in paddy water, soil, and rice straw was determined by plotting the residual level of broflanilide against time. The maximum squares of correlation coefficients were used to determine the best-fit parameters. Exponential relations were found to accurately describe the broflanilide dissipation kinetics for all samples. This was confirmed by the linearity of the plots of broflanilide level vs. time, according to the first-order kinetic equation:

(2) $$\begin{array}{c} Ct=C0s^{-Kt}, \end{array}$$

(3) $$\begin{array}{c} T_{\left(1/2\right)}=\frac{\ln\;2}{K}, \end{array}$$

where *Ct* (mg·kg^−1^ or mg·L^−1^) represents the concentration of the broflanilide residue at the time of *t* (days), *C*0 (mg·kg^−1^ or mg·L^−1^) represents the initial concentration after application, *K* is the first-order rate constant, and *T*_1/2_ is the half-life period of broflanilide that is calculated from the *K* value for each experiment [33].

Experimental data were analyzed using SPSS v10.0 (SPSS Inc., Chicago, USA) and Origin v9.1 (OriginLab, Stevenson, USA).

## 3. Results and Discussion

### 3.1. Optimization of the HPLC Method

#### 3.1.1. Optimization of Detection Wavelength

**Figure 2:**
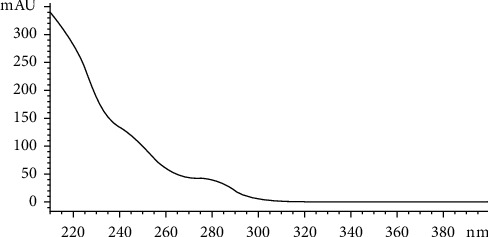
Ultraviolet absorption curve of broflanilide.

The broflanilide standard solution was scanned at 190–400 nm by HPLC to obtain the corresponding absorption wavelength and response value of the UV absorption spectra (Figure 2). The UV absorption pattern of broflanilide showed a maximum at 210 nm and a subabsorption band at 240–260 nm. When the wavelength of the UV detector was set at less than 220 nm, reagents including acetonitrile showed large UV absorption, and noise from the solvent generated obvious interference. The analysis was difficult in such cases because of poor stability. Detection at 254 nm showed little interference from the solvent, and the target compounds and impurity peaks were separated clearly. Therefore, 254 nm was considered optimal for the HPLC analysis of broflanilide.

#### 3.1.2. Optimization of Chromatographic Column

The chromatographic column notably affects the retention behavior and peak shape of the analyte. Particularly, a suitable column can improve retention [34]. To minimize the running time and achieve the best peak shape, the HPLC conditions were optimized using two different C18 columns, Zorbax Eclipse XDB-C18 column (4.6 mm × 150 mm × 5 *μ*m) and Zorbax SB-C18 column (2.1 mm × 100 mm × 1.8 *μ*m). Using the same mobile phase, broflanilide had a longer retention time on the XDB-C18 column than the SB-C18 column, indicating better separation of broflanilide from the impurity peaks. The XDB-C18 column was therefore chosen for use in subsequent analysis.

#### 3.1.3. Optimization of Mobile Phase

To improve the sensitivity of HPLC, the effects of different flows on the separation of broflanilide were investigated. Three mobile phases were tested, methanol-water, acetonitrile-water, and acetonitrile-water with 0.1% formic acid. The results showed that the acetonitrile-water phase yielded a shorter separation time and narrower peaks than the methanol-water phase. Moreover, acetonitrile is a stronger solvent than methanol and thus could effectively remove impurities from the column [35].

**Figure 3:**
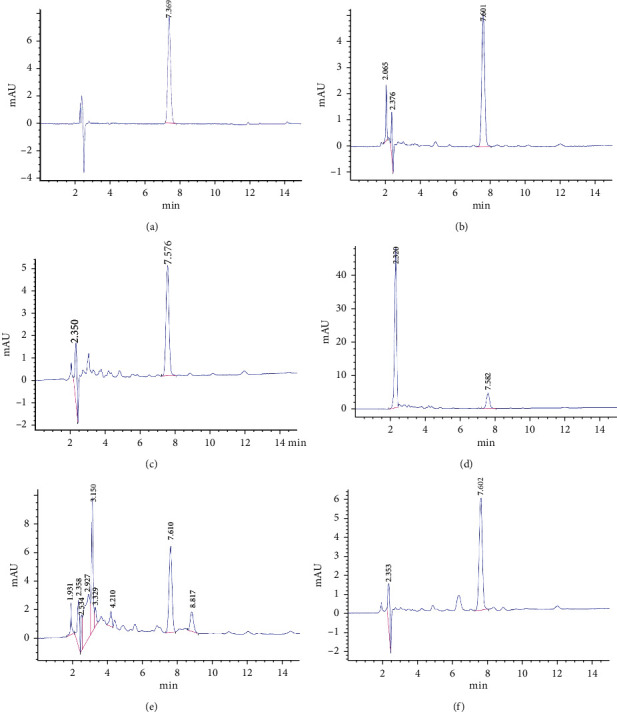
HPLC chromatograms of broflanilide detected in the standard solution (5 mg·kg^−1^); (a), paddy water (b), paddy soil (c), rice straw (d), rice husks (e), and rice grains (f). Water samples were spiked at 0.5 mg·kg^−1^, whereas soil and rice samples were spiked at 1 mg·kg^−1^.

The addition of 0.1% formic acid to the mobile phase could optimize the peak shape and thereby improve the sensitivity of HPLC [36]. However, during detection with acetonitrile-water with 0.1% formic acid as the mobile phase, an unduly low ratio of acetonitrile resulted in too long a retention time, and thereby delayed the detection of the next sample; an unduly high ratio of acetonitrile resulted in too short a retention time, and thus led to the interference of impurity peaks. When the mobile phase consisted of acetonitrile-water with 0.1% formic acid in a volume ratio of 75 : 25 (flow rate = 0.5 mL·min^−1^), the target peak and impurity peaks were clearly separated. In this case, the target peak was symmetrical, the baseline was stable, and the retention time was moderate. Therefore, acetonitrile-water with 0.1% formic acid (75 : 25, v/v) was adopted as the optimal mobile phase for HPLC analysis of broflanilide residues. The HPLC chromatograms of the standard and spiked samples are shown in Figures 3(a)–3(f).

### 3.2. Optimization of the QuEChERS Procedure

#### 3.2.1. Optimization of Solvent

**Figure 4:**
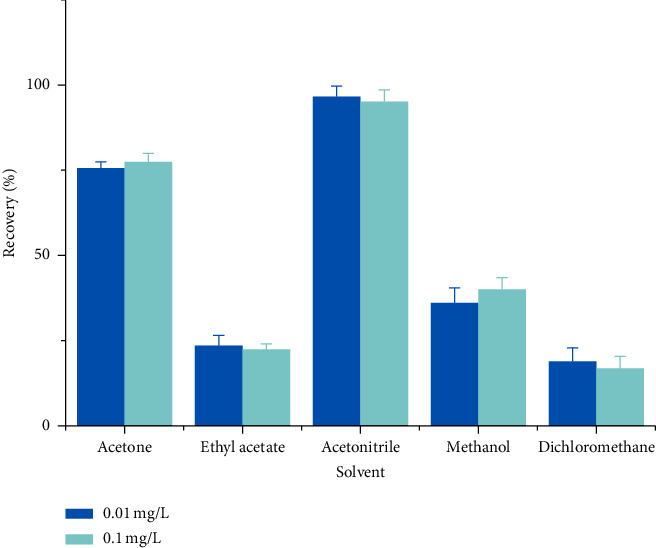
Effects of different solvents (acetone, ethyl acetate, acetonitrile, methanol, and dichloromethane) on the recovery of broflanilide in paddy water samples spiked at levels of 0.01 and 0.1 mg·L^−1^ (*n* = 3).

In insecticide residue analysis, the pretreatment and preparation of samples are the most time-consuming and complicated procedures. The chemical properties of the insecticide determine which solvents can be used for its extraction: typical examples include acetone [37], ethyl acetate, acetonitrile [38], methanol [39], and dichloromethane [40]. This study compared these five solvents for the recovery of broflanilide in paddy water samples, finding that acetonitrile gave the highest recovery (Figure 4). Moreover, acetonitrile is characterized by less lipid and protein interference, higher compatibility with HPLC, and fewer coextracted matrix components compared with other solvents [41].

**Figure 5:**
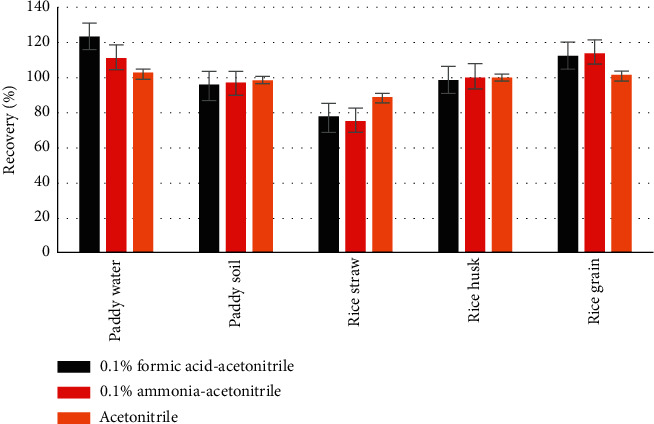
Effect of different solvent pHs on the recovery of broflanilide in various matrices (*n* = 3): (a) Matrices spiked at 0.1 mg·kg^−1^; (b) Matrices spiked at 0.5 mg·kg^−1^.

Then, a certain volume fraction (0.1%) of formic acid and sodium hydroxide (NaOH) was added to acetonitrile, and the recovery of broflanilide in five different matrices that spiked at levels of 0.1 and 0.5 mgkg^−1^ was investigated to optimize the extraction. The results showed that the recovery of broflanilide from paddy soil and rice hulls samples was not significantly affected by adding acid or alkali to the acetonitrile. Among the remaining three matrices, recovery was best using acetonitrile without acid or alkali, with the recovered amounts of broflanilide being closest to the added amount (Figure 5). Therefore, acetonitrile without acid or alkali was selected as the extraction solvent for sample pretreatment.

#### 3.2.2. Optimization of Sorbent

**Figure 6:**
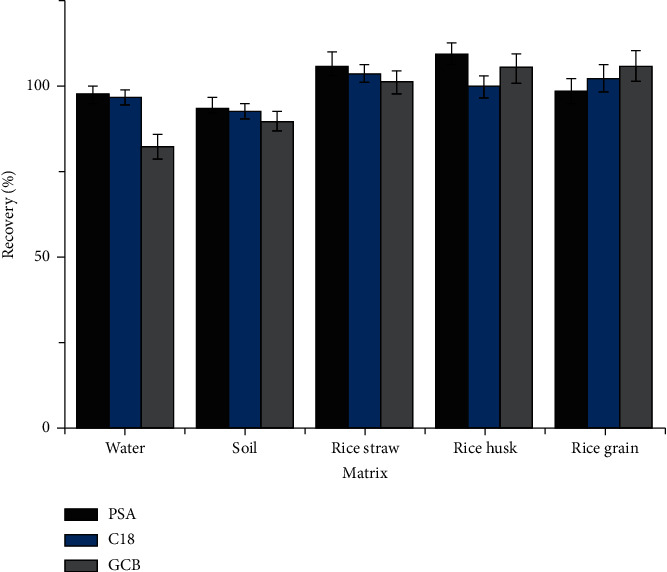
Effects of different sorbents (PSA, N-propylethylenediamine; C18, octadecylsilane; and GCB, graphitized carbon black) on the recovery of broflanilide in different matrices spiked at the level of 0.1 mg·L^−1^ (or mg·kg^–1^; *n* = 3).

To acquire satisfactory extractions of broflanilide, PSA, C18, and GCB were compared as sorbents in the QuEChERS procedure. PSA is a weak anion exchanger that extracts organic acids and carbohydrates from the matrix [42], C18 is a nonpolar material used for removal of nonpolar and moderately polar compounds in polar samples, and GCB is an effective sorbent for removing pigments, including chlorophyll and carotenoids [43]. The use of MgSO_4_ and NaCl for partitioning could yield a substantial volume of the upper layer and give high recoveries [44]. Herein, the extraction of broflanilide was conducted using 50 mg of each specific sorbent (0.1 mg·kg^−1^) and 150 mg of MgSO_4_. The average recoveries of broflanilide in various matrices ranged from 89.67% to 109.35% for the different sorbents (Figure 6), which met the requirements (70%–120% recovery) of the Agricultural Industry Standard of the People's Republic of China [45].

**Figure 7:**
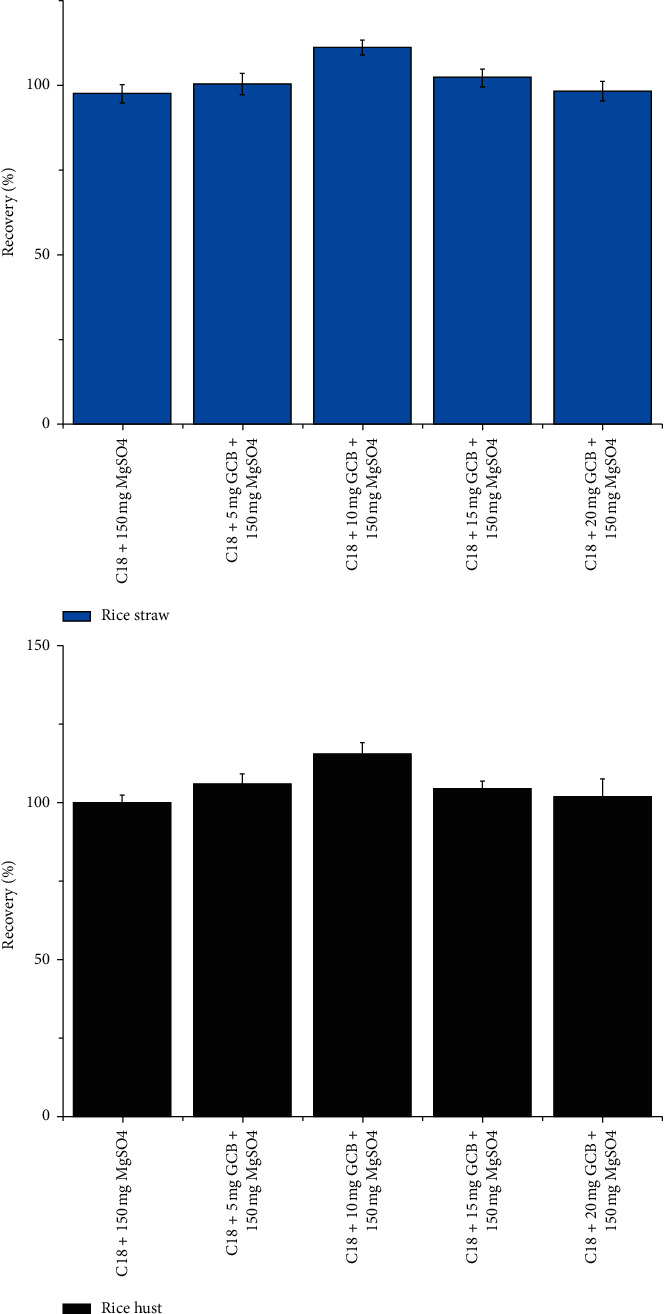
Effects of different sorbent dosages (5, 10, 15, and 20 mg of GCB) on the recovery of broflanilide in rice straw and husks spiked at the level of 0.1 mg·kg^–1^ (*n* = 3).

Because rice straw and husks had high contents of pigments, C18 was combined with GCB to reduce interference. Given the high sorption capacity of GCB for pigments [46], it is critical to control the sorbent dosage to prevent insufficient recovery. The effects of different dosages of GCB (5, 10, 15, and 20 mg) on the recovery of broflanilide were compared (Figure 7), and 10 mg of GCB was considered optimal for broflanilide extraction.

In summary, the optimal conditions for the extraction and purification of broflanilide by the QuEChERS procedure were obtained: 50 mg PSA + 150 mg MgSO_4_ for paddy water, 50 mg C18 + 150 mg MgSO_4_ for paddy soil and rice grains, 50 mg C18 + 10 mg GCB + 150 mg MgSO_4_ for rice straw and husks.

### 3.3. Validation of the QuEChERS-HPLC Method

#### 3.3.1. Specificity, Linearity, LOD, LOQ, and Matrix Effect

No interference was detected in blank samples of different matrices during the retention time, indicating a high specificity of the proposed method for broflanilide. Least-squares linear regression gave six-point calibration plots for broflanilide (0.005, 0.01, 0.05, 0.1, 0.5, and 1 mg·kg^−1^) that exhibited good linear relationships in all matrices (correlation coefficient >0.99). The LODs for broflanilide ranged from 0.16 to 1.67 *μ*g·kg^−1^ while the LOQs ranged from 0.54 to 5.48 *μ*g·kg^−1^ in the different matrices (Table 1). The matrix effects for broflanilide ranged from −69% to −54% (Table 1), indicating matrix suppression [47]. The results show that all the matrices spiked with broflanilide significantly suppressing the response of the instrument. In other words, broflanilide had a signal suppression effect in all matrices.

#### 3.3.2. Accuracy and Precision

The average recoveries of broflanilide in paddy water ranged from 90.58% to 96.24%, with RSD of 3.37%–6.51%. The recoveries of broflanilide in paddy soil were between 87.70% and 92.91%, with RSD of 5.49%–7.51%. The recoveries of broflanilide in rice straw, husks, and grains were 85.82%–93.69% (RSD = 3.74%–8.15%), 90.00%–96.41% (RSD = 3.29%–5.24%), and 89.42%–97.46% (RSD = 4.62–6.82%), respectively (Table 2). The recoveries were in the acceptable range of 70%–110%, and all the RSD values were below 20%. Therefore, the technique meets the requirements of the Agricultural Industry Standard of the People's Republic of China (70%–120% recovery). The results indicate that the proposed method could achieve satisfactory accuracy and precision for the detection of broflanilide residues in rice and related environmental samples.

#### 3.3.3. Field Application

Based on the optimized QuEChERS-HPLC method, the residual broflanilide concentrations in 100 actual samples were determined. The concentration levels ranged from 0.95 to 14.86 *μ*g·kg^−1^, which were lower than the maximum allowable residue limit of 0.05 mg·kg^−1^ specified in the national food safety standard of China [48]. Therefore, the proposed method could be applied to rice and its related environment for the determination of broflanilide residues.

### 3.4. Dissipation Dynamics

#### 3.4.1. Paddy Water Samples

**Figure 8:**
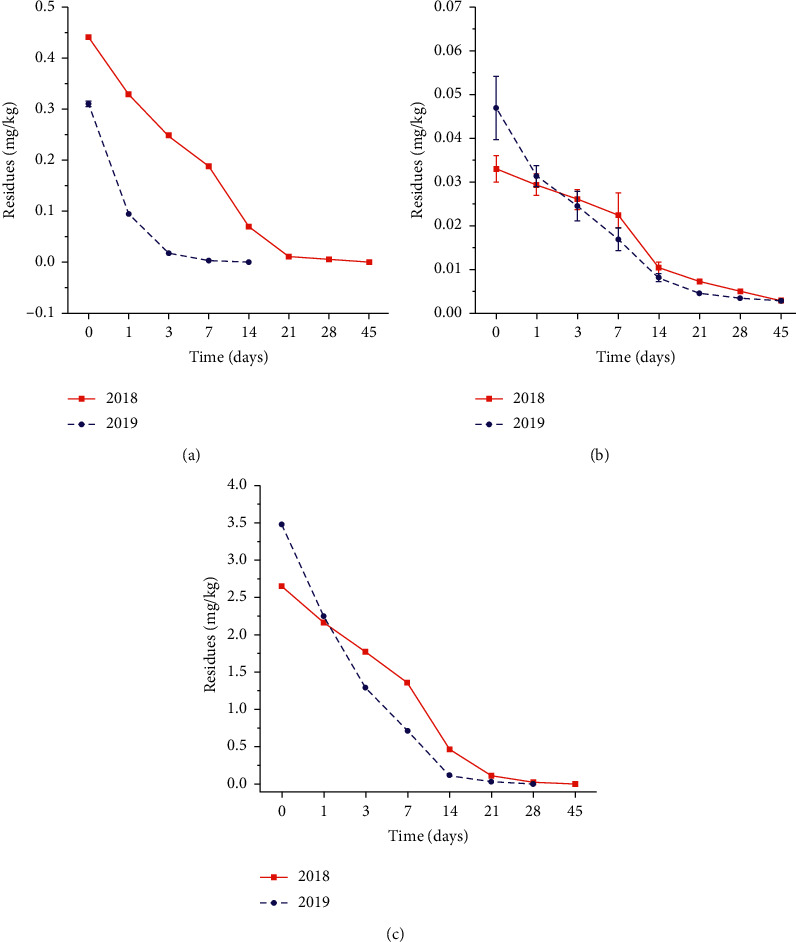
The dissipation curve of broflanilide in paddy water (a), paddy soil (b), rice straw (c) in two consecutive years (2018 and 2019).

The dissipation data results for paddy water are shown in Figure 8(a). The initial residual levels of broflanilide in paddy water samples treated with the 45 g a.i·ha^−1^ at 2 h after application were 0.4416 mg·kg^−1^ in 2018 and 0.3115 mg·kg^−1^ in 2019. Figure 8(a) shows that broflanilide dissipated rapidly in paddy water from 2 h to 3 days and then decrease afterward, and the residues of broflanilide could not be quantified accurately in paddy water 21 days after application in 2019. This indicated that the degradation half-life of broflanilide was less than 3 days in paddy water. The dissipation dynamics of broflanilide in paddy water samples could be described by first-order kinetics equations (Table 3). The initial residual levels and the *T*_1/2_ of broflanilide in 2018 were higher than in 2019, that may be related to the dilution by rainfall [49], different temperature, and other factors.

#### 3.4.2. Paddy Soil Samples

The dissipation data results for paddy soil are shown in Figure 8(b). The initial residual levels of broflanilide in paddy soil samples treated with the 45 g a.i·ha^−1^ at 2 h after application were 0.0329 mg·kg^−1^ in 2018 and 0.0468 mg·kg^−1^ in 2019. Figure 8(b) shows that the dissipation of broflanilide in paddy soil mainly occurred during 2 h to 14 days after field application, and the half-life of broflanilide was less than 6 days in paddy soil. The dissipation dynamics of broflanilide in paddy soil samples could be described by first-order kinetics equations (Table 3). The dissipation of pesticides in soil depends not only on the pesticide but also on the soil properties, including organic matter content, climate conditions, and microbial activity [50]. The initial residual levels of broflanilide in two years were similar, but the *T*_1/2_ of broflanilide in 2018 was about twice that of 2019, which may largely be due to relatively high soil organic carbon content in soil samples in 2018.

#### 3.4.3. Rice Straw Samples

The dissipation data results for rice straw are shown in Figure 8(c). The initial residual levels of broflanilide in rice straw samples treated with the 45 g a.i·ha^−1^ at 2 h after application were 2.6491 mg·kg^−1^ in 2018 and 3.4780 mg·kg^−1^ in 2019. Figure 8(c) shows that the dissipation of broflanilide in rice straw mainly occurred during 2 h to 14 days after field application. The dissipation dynamics of broflanilide in rice straw samples could be described by first-order kinetics equations (Table 3). The dissipation of pesticides in rice straw is usually related to the climate and experimental conditions (e.g., light and heat) and the growth dilution factor [51]. In 2019, the residual levels of broflanilide were observed to decrease rapidly in the period from 2 h to 3 days. Statistics indicated that the average temperature was higher in 2019 than 2018. This explains why the half-life of broflanilide in 2019 was much shorter than that in 2018. Therefore, the climate conditions may play an important role in the half-life of broflanilide in rice straw.

### 3.5. Terminal Residues

**Figure 9:**
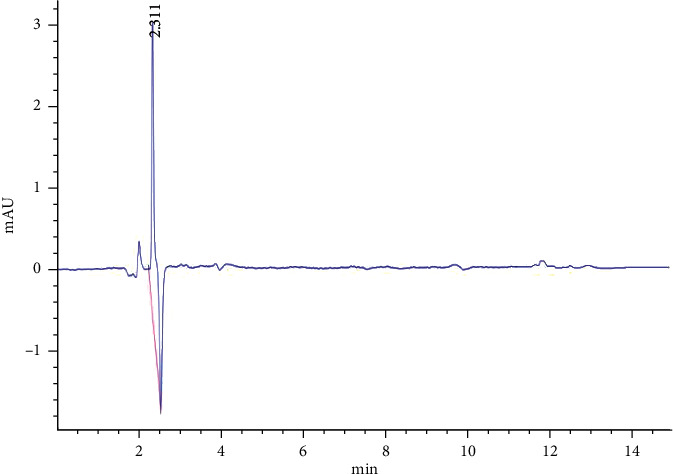
HPLC chromatogram of rice husk samples obtained during harvest time in the experimental field applied at 45 g a.i·ha^−1^.

Paddy soil, rice straw, rice husk were sampled and stored during harvest from the plots treated with broflanilide 5% SC at the recommended dose and at a dose 1.5-fold higher than the recommended dose, respectively. The residues of broflanilide in paddy soil, rice straw, and rice husk samples were not detectable or were below LOQ at harvest (Figure 9).

## 4. Concluding Remarks

This study developed an effective method for the determination of broflanilide residues and investigated the dissipation dynamics in paddy water, paddy soil, and rice straw from a paddy field. The proposed method involved an optimized QuEChERS procedure for sample pretreatment followed by HPLC analysis of broflanilide residues. Satisfactory results were achieved in terms of specificity, linearity, analytical limits, accuracy, and precision. Moreover, the method not only saves time and effort but can also reduce the use of organic solvents. The initial residual levels of broflanilide in paddy environments increased in the order paddy soil < paddy water < rice straw. The dissipation dynamic tests indicated broflanilide half-lives of 0.46–2.46, 2.09–5.34, and 1.31–3.32 days in soil, water, and rice straw, respectively. Broflanilide was dissipated more rapidly in water than in soil and rice straw. According to the terminal results, the residues of broflanilide in paddy soil, rice straw, rice husk samples were not detectable or were below LOQ at harvest. Our findings suggest that broflanilide could be safely used in rice at the recommended dosage. The developed method can thus be applied for regular monitoring of broflanilide residues in practical rice production and provides a useful tool for the analysis of broflanilide residues in other media. This research could provide guidance on the proper and safe use of this pesticide in agricultural products and the environment.

## Figures and Tables

**Table 1:** Regression equations, correlation coefficients (*R*^2^), limits of detection (LODs), limits of quantification (LOQs), and matrix effects for broflanilide in different matrices.

Matrix	Regression equation	*R* ^2^	LOD (mg·kg^−1^)	LOQ (mg·kg^−1^)	Matrix effect
Acetonitrile	*y* = 18.4981*x* + 1.5376	0.9954	–	–	–
Paddy water	*y* = 7.6045*x* + 0.1697	0.9920	0.00016	0.00054	−59%
Paddy soil	*y* = 7.8894*x* + 0.1364	0.9976	0.00084	0.00251	−57%
Rice straw	*y* = 6.8757*x* + 0.2101	0.9981	0.00148	0.00526	−63%
Rice husks	*y* = 5.8066 + 0.1837	0.9934	0.00167	0.00548	−69%
Rice grains	*y* = 8.5683 + 0.1420	0.9926	0.00096	0.00253	−54%

**Table 2:** Recoveries and relative standard deviations of broflanilide in different matrices spiked at three concentration levels each (*n* = 5).

Matrix	Spiked level (mg·kg^–1^)					Recovery (%)	Average recovery (%)	Relative standard deviation (%)
	1	2	3	4	5			
Paddy water	0.01	92.17	86.51	100.28	98.04	87.98	93.00	6.51
	0.05	101.22	93.45	95.77	97.35	93.40	96.24	3.37
	0.5	95.27	84.36	87.23	92.54	93.48	90.58	5.06

Paddy soil	0.1	89.36	80.02	92.54	86.46	90.14	87.70	5.49
	0.5	82.79	94.41	99.37	98.80	89.20	92.91	7.51
	1	89.73	80.54	94.64	86.15	88.45	87.90	5.86

Rice straw	0.1	80.62	86.07	88.93	87.90	85.57	85.82	3.74
	0.5	87.08	92.35	98.25	101.30	89.20	93.64	6.42
	1	95.30	102.11	90.27	98.34	82.45	93.69	8.15

Rice husks	0.1	96.32	94.68	92.39	100.75	97.92	96.41	3.29
	0.5	88.28	101.51	91.71	94.03	92.41	93.59	5.24
	1	94.30	88.75	85.19	91.40	90.34	90.00	3.74

Rice grains	0.1	80.65	88.81	88.72	92.43	96.48	89.42	6.54
	0.5	96.36	97.63	104.52	92.03	96.78	97.46	4.62
	1	90.61	79.25	93.48	94.25	92.15	89.95	6.82

**Table 3:** Dissipation dynamics equation of broflanilide in paddy field.

Samples	Time	First-order kinetic equation	*R* ^2^	*T*_1/2_
Paddy water	2018	*Ct* = 0.5829*e*^−0.2818*t*^	0.9998	2.46
	2019	*Ct* = 1.6234*e*^−1.5094*t*^	0.9974	0.46
Paddy soil	2018	*Ct* = 0.0377*e*^−0.1298*t*^	0.9953	5.34
	2019	*Ct* = 0.0639*e*^−0.3322*t*^	0.9925	2.09
Rice straw	2018	*Ct* = 3.3488*e*^−0.2208*t*^	0.9947	3.32
	2019	*Ct* = 6.1737*e*^−0.5307*t*^	0.9954	1.31

## Data Availability

The data used to support the findings of this study were supplied by Jiangxi Veterinary Drug and Feed Supervision Institute under license and so cannot be made freely available.

## Abbreviations

C18: Octadecylsilane
GCB: Graphitized carbon black
HPLC: High-performance liquid chromatography
QuEChERS: The quick, easy, cheap, effective, rugged, and safe method
SPME: Solid phase microextraction
MSPD: Matrix solid phase dispersion
PSA: *N*-propylethylenediamine
MRL: Maximum residue limit.
